# Conformational Sampling and Nucleotide-Dependent Transitions of the GroEL Subunit Probed by Unbiased Molecular Dynamics Simulations

**DOI:** 10.1371/journal.pcbi.1002004

**Published:** 2011-03-10

**Authors:** Lars Skjaerven, Barry Grant, Arturo Muga, Knut Teigen, J. Andrew McCammon, Nathalie Reuter, Aurora Martinez

**Affiliations:** 1 Department of Biomedicine, University of Bergen, Bergen, Norway; 2 Computational Biology Unit, Bergen Center for Computational Science, University of Bergen, Bergen, Norway; 3 Department of Chemistry and Biochemistry, Center for Theoretical Biological Physics, University of California San Diego, La Jolla, California, United States of America; 4 Howard Hughes Medical Institute, University of California San Diego, La Jolla, California, United States of America; 5 Biophysics Unit (CSIC/UPV) and Department of Biochemistry and Molecular Biology, University of the Basque Country, Bilbao, Spain; 6 Department of Pharmacology, University of California, San Diego, La Jolla, California, United States of America; 7 Department of Molecular Biology, University of Bergen, Bergen, Norway; Baylor College of Medicine, United States of America

## Abstract

GroEL is an ATP dependent molecular chaperone that promotes the folding of a large number of substrate proteins in *E. coli*. Large-scale conformational transitions occurring during the reaction cycle have been characterized from extensive crystallographic studies. However, the link between the observed conformations and the mechanisms involved in the allosteric response to ATP and the nucleotide-driven reaction cycle are not completely established. Here we describe extensive (in total 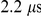 long) unbiased molecular dynamics (MD) simulations that probe the response of GroEL subunits to ATP binding. We observe nucleotide dependent conformational transitions, and show with multiple 100 ns long simulations that the ligand-induced shift in the conformational populations are intrinsically coded in the structure-dynamics relationship of the protein subunit. Thus, these simulations reveal a stabilization of the equatorial domain upon nucleotide binding and a concomitant “opening” of the subunit, which reaches a conformation close to that observed in the crystal structure of the subunits within the ADP-bound oligomer. Moreover, we identify changes in a set of unique intrasubunit interactions potentially important for the conformational transition.

## Author Summary

Molecular machines convert chemical energy to mechanical work in the process of carrying out their specific tasks. Often these proteins are fueled by ATP binding and hydrolysis, enabling switching between different conformations. The ATP-dependent chaperone GroEL is a molecular machine that opens and closes its barrel-like structure in order to provide a folding cage for unfolded proteins. The quest to fully understand and control GroEL and other molecular machines is enhanced by complementing experimental work with computational approaches. Here, we provide a description of the molecular basis for the conformational changes in the GroEL subunit by performing extensive molecular dynamics simulations. The simulations sample the conformational population for the different nucleotide-free and bound states in the isolated subunit. The results reveal that the conformations of the subunit when isolated resemble those of the subunit integrated in the GroEL complex. Moreover, the molecular dynamics simulations allow following detailed changes in individual interatomic interactions brought about by ATP-binding.

## Introduction

GroEL participates in the folding of 5-10% of cellular *Escherichia coli* proteins by providing an isolated chamber for non-native substrate proteins together with a heptameric ring shaped co-chaperonin, denoted GroES [1]–[5]. It has also been suggested that GroEL might forcefully unfold kinetically trapped misfolded intermediates [6], [7]. GroEL is composed of two heptameric rings made up of identical subunits, each of 57 kDa. The two separate rings, denoted cis (active) and trans (inactive), are stacked back-to-back to form two folding environments (or cages) working off-phase, analogous to a two-stroke engine. Each subunit is divided into three domains; equatorial (residues 1-133, 409-548), intermediate (134-190, 377-408), and apical (191-376) domain, separated by two hinges that facilitate large conformational transitions in the complex [8]–[10].

Substantial mechanistic insight has been obtained through comparison of the large amount of structural data available for the GroEL complex. The first high-resolution X-ray structure was released in 1994 by Braig *et al.* [8], and since then, numerous structural studies, including X-ray crystallography, cryo-EM, and NMR have been published of different functional states of GroEL (for reviews see [11], [12]). On this structural background it has been possible to make predictions and educated hypothesis about the transition pathways during the protein functional cycle. This includes the ATP dependent opening of the cis cavity with the concomitant increase of its volume from 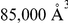 to 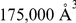 [10]. The conformational transitions occurring on the subunit level are substantial, and its trajectory is generally explained in a sequential manner.

Binding of ATP to each of the seven equatorial domains in the *cis* ring, together with a non-native polypeptide produces a 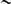 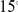 counterclockwise twist of the apical domain, and a downward rotational movement of the intermediate domain [13], [14]. This structural state of the ring is denoted **R** (where each of the 7 subunits are in the **r** state) while the initial closed state is denoted **T** (each of the 7 subunits are in the **t** state). Reaching the **R** state facilitates GroES association to the apical domains of the cis ring promoting much larger conformational changes and resulting in the fully open **R′** conformation (all seven subunits in the **r′** state). This **r′** conformer is characterized by a 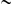 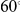 elevation and 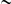 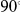 clockwise twist of the apical domains (opposite direction to that seen upon ATP binding) [10]. Non-native polypeptide folding takes place within the *cis* ring of the **R′** state of the reaction cycle, which is the longest lived (about 8–10 s) [15], and continues until ATP hydrolysis induce the **R″** conformation permiting ATP binding to the opposite *trans* ring [16], [17]. This final rearrangement result in a conformer very similar to the **r′** form (RMSd of 1.46 Å).

Despite this extensive structural insight, the mechanisms involved in allosteric signaling are not yet fully understood at atomic and residue level. In general, X-ray crystallography provides invaluable snapshots of different states of the protein reaction cycle, but not of the transitions between them. In this context, computational approaches have the potential to nicely complement the experimental techniques. In particular, molecular dynamics (MD) simulations provide important insight into protein dynamics at the atomic level, and allow following subsequent individual atomic interactions and fluctuations as a function of time [18]. Additionally, normal mode analysis (NMA) has proven to be efficient and accurate in the task of predicting and describing large scale conformational transitions in proteins [19]–[22]. NMA analytically characterizes all possible deformations of a protein around a stable equilibria with respect to their energetic cost. Although the utilization of NMA involves a loss of time-dependent fluctuations and resolution, i.e. by the use of coarse-grained models, it has been shown that it provides functionally relevant motions, and information on allosteric mechanisms [23]–[28].

Various computational methods have indeed provided essential information on the GroEL subunit dynamics. Notably, the transitions between the main functional states (**T**, **R**, and **R″**) have been further refined by utilizing NMA [27]–[29], targeted MD (TMD) [30], brownian dynamics [31], principal component analysis (PCA) [32], and MD simulations [33], [34]. Moreover, a number of computational studies have been dedicated to find pathways for both intra- and inter-ring communication in GroEL [35]–[40]. From these, several residues have been pointed out as important for the allosteric signaling thus increasing the understanding of the involved mechanisms.

The concept of the two-stage transition has been strengthened by the TMD simulation of the GroEL subunit which pulls the **t** form to the fully open **r″** form along a 500 ps long MD simulation [30]. This computational study suggested that the transition begins with a downward tilt of helix M, and the subsequent counterclockwise twist of the apical domain. Moreover, biasing the MD simulation by employing temperature acceleration to increase the conformational sampling recently showed the ability of the isolated GroEL subunit to undergo the **t** to the **r″** state transition [34]. However, this simulation was not able to sample the closing of the binding pocket as seen in the X-ray structures of the **r″** conformers. Finally, unbiased MD simulation of the GroEL subunit has been performed in order to investigate the ATP-driven conformational changes [33]. This simulation samples the transition from the **t** to the semi-relaxed **r** conformation during 20 ns, nicely illustrating formation and rupture of hydrogen bonds.

A full scale monitoring of individual particle motions to probe the mechanistic basis for the transitions requires extensive unbiased conformational sampling at atomistic resolution. The 20 ns long MD simulations of Sliozberg and Abrams are too short to observe the full scale transitions of the subunit [33]. Moreover, significant variations between individual MD simulations have been detected [23], [41], [42], thus highlighting the importance of multiple simulations to extract statistically relevant information on the conformational changes. Investigating the positive (intra-ring) and negative cooperativity (inter-ring) of ATP-binding would require MD simulations of the entire GroEL oligomer. Such simulations on relevant timescales for conformational transitions are beyond the capabilities of present computational resources. It has also been reported the surprising facility to obtain stable folded monomers of GroEL by a variety of means [43]–[47], which can be explained by the small area buried by the monomers at subunit interfaces in the X-ray structure of the GroEL complex [8]. Furthermore, under some particular experimental conditions it seems that monomeric GroEL exhibits a weak chaperone activity [46]. Despite the fact that the chaperone activity of monomeric GroEL is a controversial issue [48], the ability of the monomer to fold into a native-like conformation that can bind nucleotide, points to the subunit as a relevant structural unit to be investigated by MD simulations.

In the current work we present extensive (in total 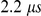 long) unbiased MD simulations of the GroEL subunit starting from the closed (**t**) and open (**r″**) conformations, with and without bound nucleotide. We use PCA of 287 experimentally obtained GroEL subunits to interpret the conformational sampling of our simulations. We observe nucleotide dependent shifts in the conformational ensembles, and show that the subunit response, as observed in the oligomeric structure, is intrinsically coded in the structure-dynamics of the isolated subunit. These simulations provide so far unexplored sampling from **t** all the way to a structure close to **r″**. A nearly complete transition in the opposite direction is also sampled by removing ADP from the **r″** form. Another interesting outcome of our simulations is that the inherent motion of unliganded GroEL subunit is biased along the transition pathway towards both the **r** and **r″** states. Furthermore, the MD simulations reveal a weak stabilization of the equatorial domain upon ATP binding, resulting in a modest decrease of the configurational entropy of this domain. Conversely, a larger increase of entropy is found for the whole GroEL subunit. Finally, we decipher the underlying mechanisms for the conformational transitions by investigating the atomic interactions unique to the unliganded and nucleotide bound structures obtained from the X-ray structures and MD simulations. Several of the interactions that characterize the conformational intra-subunit effects brought about by ATP binding were not revealed in previous studies.

## Results

### Analysis of GroEL structures and dynamics

**Figure 1:**
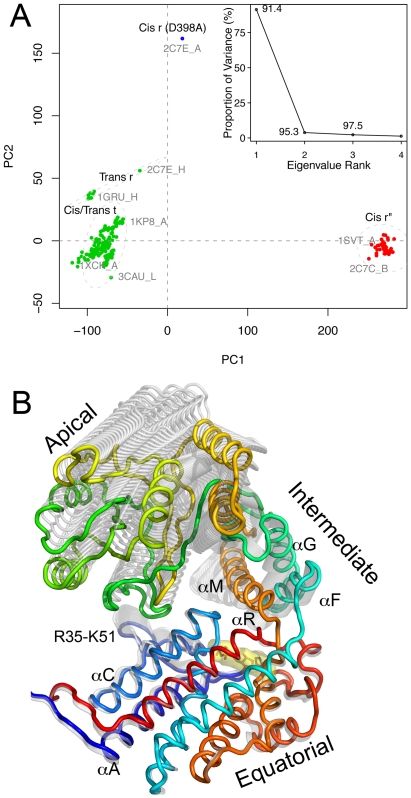
PCA of 287 GroEL subunits. (A) Experimentally obtained structures projected onto the two first principal components (PC). The inset shows the eigenvalue spectrum representing the percentage of the total variance captured by the corresponding eigenvector. Labels besides each point indicate the cumulative sum of the total variance accounted for in all lower number eigenvectors. (B) Visualization of PC1 of the X-ray structures, which consists of a downward tilt and rotation of the apical domain, along with a downward tilt of helix M in the intermediate domain. Internal helix motion is also observed within the equatorial domain along with flapping loop motion (residues 36-51).

27 crystal structures of the GroEL complex, with a total of 364 subunits, were collected from the RCSB protein databank. Six of the crystal structures were not considered due to missing coordinates, which leaves 21 GroEL complexes with 287 subunits for the following analysis. These include ATP and ADP bound conformers as well as apo forms from wild type and mutant structures. The 287 GroEL subunits were superimposed onto the invariant ‘core’ defined as the area with least structural variation [49], and PCA was performed to investigate the major conformational differences between the collected structures. As much as 91.5% of the total variance of the atomic fluctuations was captured along the first principal component (PC), while 2 and 3 dimensions were necessary to capture 95.3% and 97.5%, respectively (see inset in Figure 1A).

The GroEL subunits can be divided into three major groups along the two first PCs; the closed cis/trans **t** forms, open cis **r″** forms, and the semi-relaxed **r** forms (Figure 1A). The first PC is shown in Figure 1B and describes the main differences between the **r″** and **t** conformers. The motions described by PC1 consist of (1) an upward movement of the apical domain away from the equatorial domain; (2) a small rotation of the apical domain; (3) a downward tilt of helix M (residues 386-409) and (4) a translation of helices F and G (residues 141–152 and 155–169, respectively) along their axis of inertia. This region of the intermediate domain moves down to cover the nucleotide binding site in the equatorial domain while the apical domain twists upwards. In addition, modest internal fluctuations are observed in the equatorial domain. The stem loop spanning from Arg36 to Lys51 has a flapping motion opposite to the intermediate domain, and helices A (residues 8-27) and C (residues 65-85) show a rotation relative to helices D (residues 88-107), E (residue 113-134), and R (residues 496-514). Similar internal motions in the equatorial domain are also observed in PC2. In particular, helix A (residues 8-27) undergoes a translational motion along its longitudinal axis, while both helices D (residues 88-107) and R (residues 496-514) rotate along their axis. PC2 represents the largest variation between the **t** and **r** conformers, consisting of a counterclockwise twist and a modest elevation of the apical domain. Similarly, PC3 describes a rotational motion of the apical domain together with a translational motion of helix A along its longitudinal axis.

The internal fluctuations within the equatorial domain reveal the presence of two sub-domains (helices A+C, and helices D+E+R) consistent with a previous study utilizing 35 subunit structures [32]. Moreover, it is interesting to notice that the invariant core is a relatively small part of the equatorial domain (helices E, O, and R), thus pointing out the structural variability within this domain.

### Conformational population shift as a response to nucleotide binding

To investigate the intrinsic response of nucleotide binding to GroEL we conducted 14 MD simulations of the isolated GroEL subunit starting from both ends of the reaction cycle; the closed (**t**) and open (**r″**) states, with (holo) and without (apo) bound nucleotide (ATP or ADP). Of these, we performed four 300 ns long simulations: (A) closed **t** unbound, (B) open **r″** unbound, (C) closed **t** ATP-bound, and (D) open **r″** ADP-bound. An additional ten 100 ns long simulations of the closed **t** form were also carried out: 5 (E–J) unliganded, and 5 (K–P) ATP-bound. Simulations (D) and (K) consist of the 0–100 ns interval of simulation (A) and (C), respectively.

**Figure 2:**
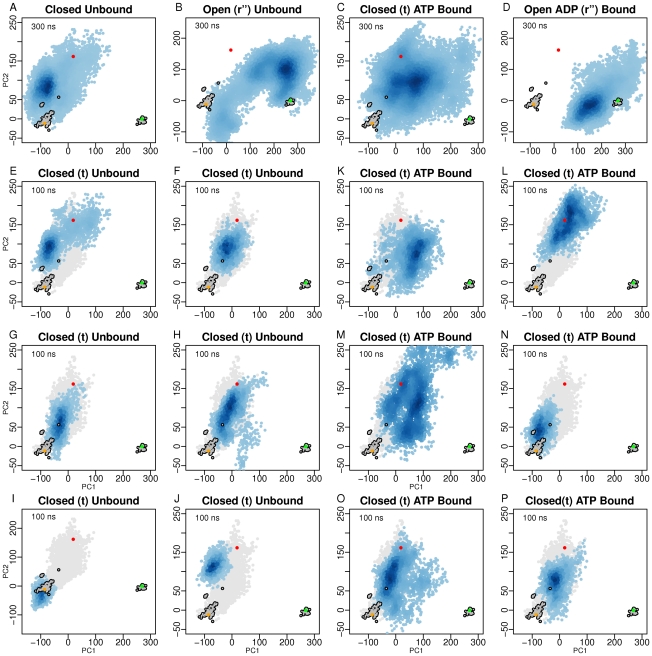
Conformer plots of X-ray and MD structures reveal the nucleotide dependent conformational sampling. Projection of X-ray (contoured grey dots) and MD (blue) structures onto the two most significant principal planes defined by 287 GroEL experimentally obtained structures. The 300 ns simulations are shown in the first row of panels, while the 100 ns simulations are shown in rows 2–4. The distribution of MD structures is depicted with density shaded blue points. Light grey dots represent the first 50 ns of the 100 ns simulations. Orange, red, and green dots represent the closed (**t**), semi-relaxed (**r**), and open (**r″**) X-ray structures, respectively. The conformer plots show a two dimensional representation of the conformational sampling in terms of conformational difference as described by the PC's.

Each conformer obtained from the MD simulations was projected onto the first two PCs determined from the X-ray structures (Figure 2). These projections display the relationship between the MD conformers in terms of the conformational differences described by the two first PCs, thus enabling interpretation of the conformational space sampled in each of the simulations.

Remarkably, the simulations of the closed subunit all sample the space along PC2, which describes the transition between the **t** and **r** form, independently of whether ATP is bound or not. The main difference between the unliganded and ATP-bound simulations lay in the sampling along PC1; the ATP bound simulations shift the ensemble of conformations along PC1, which thus comes closer to the fully open **r″** form (Figure 2A+C). This difference is most apparent in the 300 ns simulations where the ensemble samples closest to the fully open structure (see Video S1). The difference in sampling along PC1 is also significant for the 100 ns simulations (Figure S10), however they are too short to fully observe this effect. Admittedly, 100 ns is not long enough simulation time to bring the ATP bound simulation (N) away from the **t** form (Figure 2N).

A similar shift of the ensemble along PC1 and PC2 is also observed for the open simulations. Interestingly, the ADP-bound simulation samples the space in the proximity of the **r″** form (Figure 2D). Conversely, removing ADP yields a sampling of the conformational space which is significantly shifted, in particular along PC1, towards the **t** conformers (Figure 2B).

**Figure 3:**
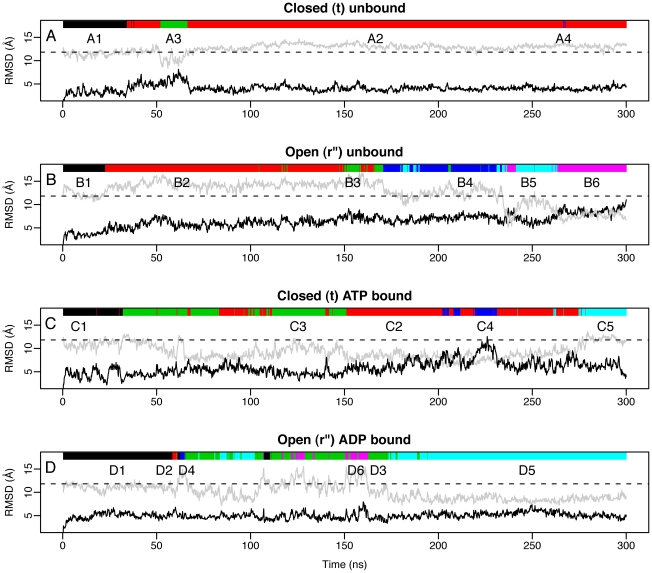
RMSd variation for the 300 ns long simulations of the GroEL subunit. Black line corresponds to the RMSd with respect to the starting structure (**t** for A+C, and **r″** for B+D), while the grey line corresponds the RMSd with respect to the opposite form (**r″** for A+C, and **t** for B+D). Each plot corresponds to one specific MD simulation; (A) closed (**t**) unbound, (B) open (**r″**) unbound, (C) closed (**t**) ATP bound, and (D) open (**r″**) ADP bound. Black dashed line depicts the RMSd value between the open (**r″**) and closed (**t**) X-ray structure. Clustering analysis is performed on each of the 4 simulations, and the bar on the top of each RMSd plot is colored according to which cluster the corresponding frame belongs to.

Clustering analysis on each of the four 300 ns long trajectories was performed to identify the predominant conformations throughout the simulations. Each frame in the trajectories is attributed to one particular cluster depicted as color bars in Figure 3 along with the resulting RMSd values. The conformations sampled in the closed apo simulation (Figure 3A) resembles its initial starting structure (**t**). Conversely, the closed holo simulation, shown in Figure 3C, comes much closer to the open **r″** form than its apo counterpart. The RMSd values for the average conformations in each of the clusters with respect to both the closed and open X-ray structures are shown in Table 1. The predominant clusters of the closed apo simulation are clusters A1+A2 which have a relatively close similarity to the closed X-ray structure, with an RMSd value of 2.56 and 3.45 Å, respectively. These low RMSd values of the closed apo simulation stand in contrast to higher RMSd values of the closed holo simulation, i.e. 5.97 and 4.09 Å for the two dominating clusters C2+C3. Cluster C4 of the closed holo simulation, which consists of 92 member conformations out of a total of 1500 MD conformers, has a higher average similarity to the open (RMSd 6.86 Å) than the closed X-ray structure (9.21 Å). We thus sample relatively close to the experimental **r″** conformer (minimal deviation of 5.8 Å) with the major difference being an open nucleotide pocket, similar to what a recent temperature biased MD simulation by Abrams and Vanden-Eijnden showed [34]. Interestingly, both the unbound and the ATP bound simulations also show a tendency to mimic the **r** form (minimal deviation of 2.6 Å) characterized by the counterclockwise rotation of the apical domain (i.e. clusters A3 and C1).

**Table 1:** Clustering analysis of MD trajectories of the GroEL subunit.

Simulation	Cluster No.	Cluster size	t	r	r″
Closed apo (A)	A1	172	2.56	4.10	11.31
	A2	1253	3.45	4.06	12.77
	A3	72	5.533	4.11	10.10
	A4	3	3.94	5.27	13.64
Open apo (B)	B1	112	12.06	11.98	3.11
	B2	664	14.14	13.57	5.65
	B3	84	14.93	13.98	7.23
	B4	269	12.52	11.79	6.80
	B5	160	10.19	9.96	6.10
	B6	211	7.23	9.36	8.11
Closed holo (C)	C1	157	4.04	3.72	10.70
	C2	654	5.97	5.62	8.10
	C3	464	4.09	5.05	9.66
	C4	92	9.21	8.94	6.86
	C5	133	5.68	4.71	11.66
Open holo (D)	D1	313	11.05	11.69	4.40
	D2	13	10.09	9.73	5.70
	D3	374	10.63	11.01	3.91
	D4	15	13.90	13.99	4.80
	D5	695	8.41	9.66	4.90
	D6	90	13.56	12.99	5.27

The RMSd values between the closed and open X-ray structures and the average conformations in each of the clusters in the four simulations of the GroEL subunit are shown. The RMSd between the open and closed X-ray structure (PDB ids: 1SVT and 1XCK, respectively) is 12.30 Å.

The RMSd values and clustering of the open (**r″**) apo and holo simulations are shown in Figure 3B+D. The apo simulation initially samples conformations around its starting point; close to the open X-ray structure. The predominant clusters of this simulation are B2 and B4 with a total of 933 member conformations, and the average conformations of these clusters have RMSd values much closer to the open than to the closed X-ray structure as shown in Table 1. After about 240 ns of simulation the structure undergoes a remarkable conformational change resulting in higher similarities towards the closed (**t**) form. These structures are assigned to cluster B6, and the member conformations have an average RMSd of 7.23 Å towards the closed X-ray, and 8.11 Å towards the open X-ray structure. We thus sample relatively close to the experimental **t** conformer starting from the **r″** conformer (minimal deviation of 5.1 Å) with the major difference being a small twist of the apical domain. Conversely, the open holo simulation consistently shows an open structure with RMSd values closer to the open **r″** than to the closed X-ray structure (Figure 3D and Table 1).

### Stabilization of equatorial domain upon nucleotide binding

**Figure 4:**
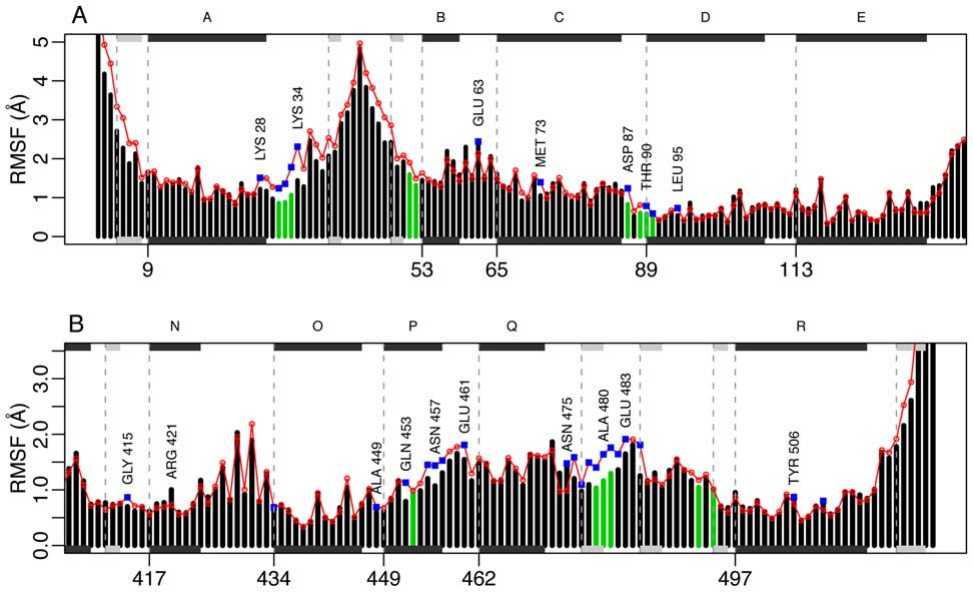
Residual fluctuations of the closed unbound and ATP bound simulations. Black bars show the RMSF values obtained from the ATP bound simulations, while red dots represent the unbound simulations. Binding site residues are depicted green, while residues that yield a significant change (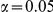) between ATP-bound and ATP-free simulations are depicted with blue squares. Residues of particular interest are labeled. RMSF values are shown for the equatorial domain (residues 2-133 (A), 409-525 (B)). Secondary structure elements are indicated schematically with helices in black and strands in gray.

To probe the differences between the unliganded and ATP-bound simulations we investigated the residue fluctuations based on the 10–60 ns interval of the subunit simulations in the closed form. As expected, most residues show a similar fluctuation pattern in the holo and apo simulations. In the equatorial domain, residues adjacent to ATP show the most evident differences between the apo and holo simulations (see Figure 4A–B). In particular, residues Lys28, Lys34, Asp87, Asn457, Glu461, Tyr478-Glu483, and Tyr485, and its immediate neighbors are significantly (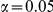) stabilized in the presence of ATP. Conversely, Glu61, Glu63, Arg421, and Asn475, show increased fluctuations in the holo simulations indicating rearrangement of these residues upon ligand binding. Modest differences in the fluctuation profiles are also observed within the apical (Figure S11A) and intermediate (Figure S11B–C) domains. Only two residues are shown to be significantly altered (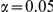); Arg350 (apical) and Lys390 (intermediate).

**Figure 5:**
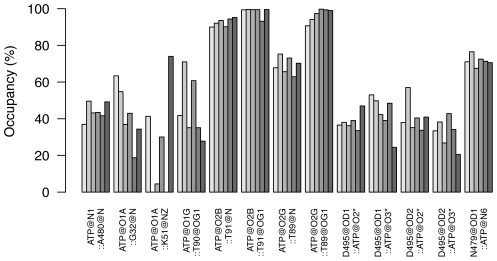
Schematic representation of potential hydrogen bonds between protein and nucleotide. Hydrogen bond donors and acceptors are labeled below the bars in which the height represents the occupancy in % of the simulation time. Each bar depicts the occupancy of one H-bond in one of the 6 ATP-bound simulations (simulations K-P in Figure 2).

Since the equatorial domain possesses the ATP binding site it is thus the epicenter for the initiation of the conformational changes. In order to relate the differences in fluctuations to binding of ATP we calculated the potential hydrogen bonds between ATP and the protein along the simulations. Figure 5 shows the occupancy of each of the ATP hydrogen bonds in the closed holo simulations. Three hydrogen bonds are shown to be particularly strong with an occupancy of more than 90% of the simulation; Thr89 and Thr91 bind to the beta and gamma phosphate, respectively. The fifth single strongest bond is Asn479 which binds to the 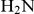 group of the adenine ring, with a occupancy of about 80% of the simulation. The negatively charged carboxyl group of Asp495 forms 4 alternating hydrogen bonds with the ribose OH, which have occupancies of approximately 40% each. Weaker bonds also exist, such as Gly32, Lys51, Thr90, and Ala480. Moreover, Asp87 is held in close contact with ATP through the tight binding to 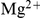, resulting in a mean distance of 3.58 Å (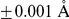) between the oxygens of Asp87 and the three adjacent phosphate oxygens of ATP.

The identified hydrogen bonds between ATP and specific protein residues can be directly linked to the change in the fluctuation pattern observed in Figure 4. In particular hydrogen bonds involving Gly32, Thr89-Thr91, and Asn479-Ala480 might cause the stabilization of these areas. Moreover, the tight binding between Asp87 and 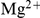 might be a stabilizing factor for Asp87 and its neighbors.

**Figure 6:**
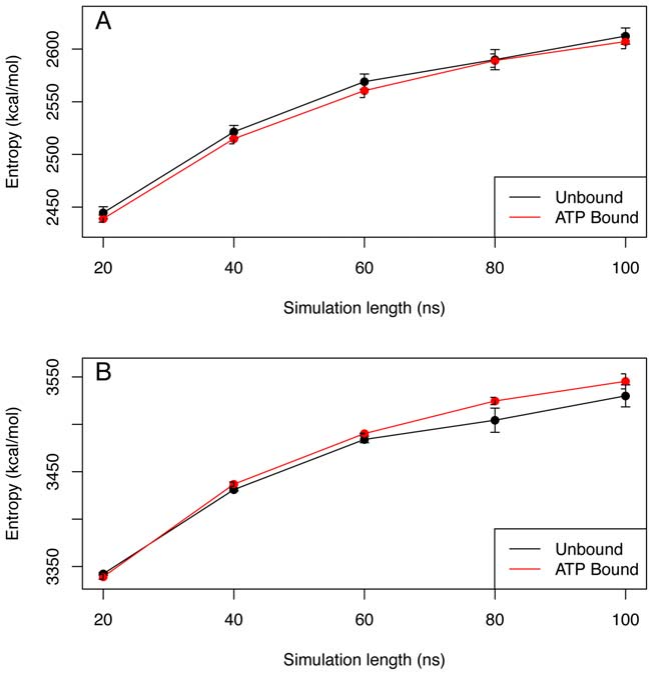
Configurational entropy. Configurational entropy of the equatorial domain (A) and the whole subunit (B) with respect to the simulation length. The calculation is based upon the 100 ns long simulations of the closed GroEL subunit; unbound (black), and ATP-bound (red).

Configurational entropies of the closed simulations were estimated to investigate the entropic penalty upon ATP binding. Entropy values calculated from quasi-harmonic analysis of MD simulations are sensitive to simulation length and the number of frames in which the calculation is based on [50]. Moreover, significant variation between individual MD has been reported [41], [51]. We thus performed entropy calculations on the multiple closed MD simulations for the equatorial domain and for the whole subunit (Figure 6). We observe only small changes in entropy within the equatorial domain upon nucleotide binding. Moreover, during the first 60 ns of the simulations, no entropy difference is found for the whole subunit, while after 80–100 ns the entropy of the ATP-bound simulation is slightly higher than that of the apo simulations.

### Differential atomic interactions

**Figure 7:**
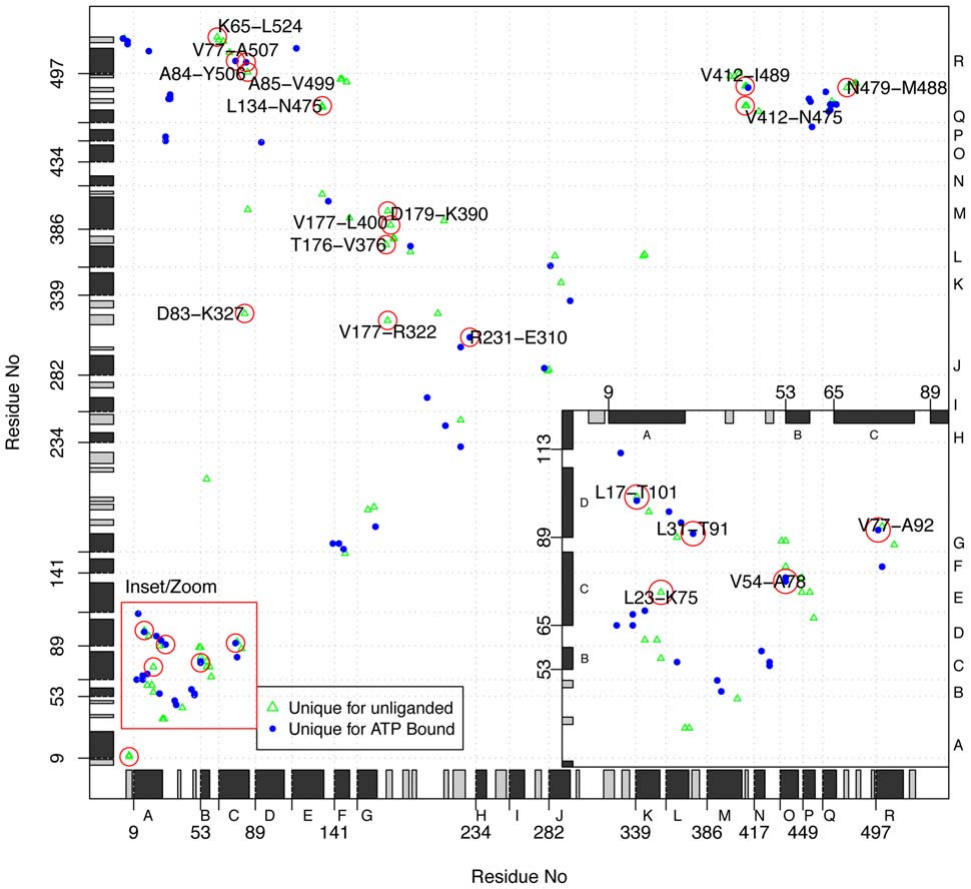
Difference contact map between ATP-bound and unliganded MD simulations. The differences in side chain contacts are mapped for the MD conformers. Two residues are assumed to be in contact if the minimal distance between them 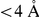. Only contacts existing in at least half of the structures are considered. Green triangles represent contacts present in the unliganded forms and not in the liganded forms. While blue dots represents contacts which are present in the liganded forms, but not in the unliganded forms. Red circles depict contacts which are found in both the X-ray and MD analysis. Secondary structure elements are indicated schematically with helices in black and strands in gray.

Comparing distance maps between different conformations has the potential of revealing unique atomic interactions potentially important for the conformational transitions within the GroEL subunit. We used difference contact maps (DCMs) to identify atomic interactions (all heavy atoms) unique to the **t**, **r**, and **r″** X-ray conformers (Figure S12A-E) complemented with DCMs obtained from the MD simulations of the closed form (Figure 7). Of particular interest are those residues which change interaction partner during the transition from **t** to **r″**.

A large number of contacts are found to be unique for either the **t** or the **r″** forms (Figure S12A). High density of differential atomic interactions are observed within the equatorial domain, and in particular the interaction pattern between helices B, C, and D is altered upon the **t**-**r″** transition. Contacts between helices B-C and C-D are found to be unique for the **r″** conformers. Conversely, the **t** conformers show tighter interactions between helix C and the loop connecting helix C and D. Here, Asn82 contacts with Asp87 in the **t** conformers but with Arg58 in the **r″**. Moreover, Asp87 changes its contact partners from Asn82 in the **t** state to Ser151 and Asp398 in the **r″** state, which is the main interaction between helix M of the intermediate domain and the equatorial domain.

Unique for the **r″** forms is also a set of hydrophobic interactions between helices C and D (Val74-Ile100, Val77-Ala96, Ala81-Ala92, Val77-Ala92). Conversely, the **t** forms have unique contacts between helix C and R (Val74-Val510, Val77-Tyr506, Ala85-Val499).

The intermediate domain also undergoes conformational rearrangements during the transition. Residues downstream of helix G initially interact with residues of helix M in the **t** form, but change interaction with helix L and K during the transition, e.g. the interaction Glu172-Arg404 in the **t** forms is changed to Glu172-Arg350 in the **r″** forms. Moreover, Asp179 contacts Lys390 at the intermediate domain in the **t** forms, and the Thr48 at the equatorial domain in the **r″** forms.

**Figure 8:**
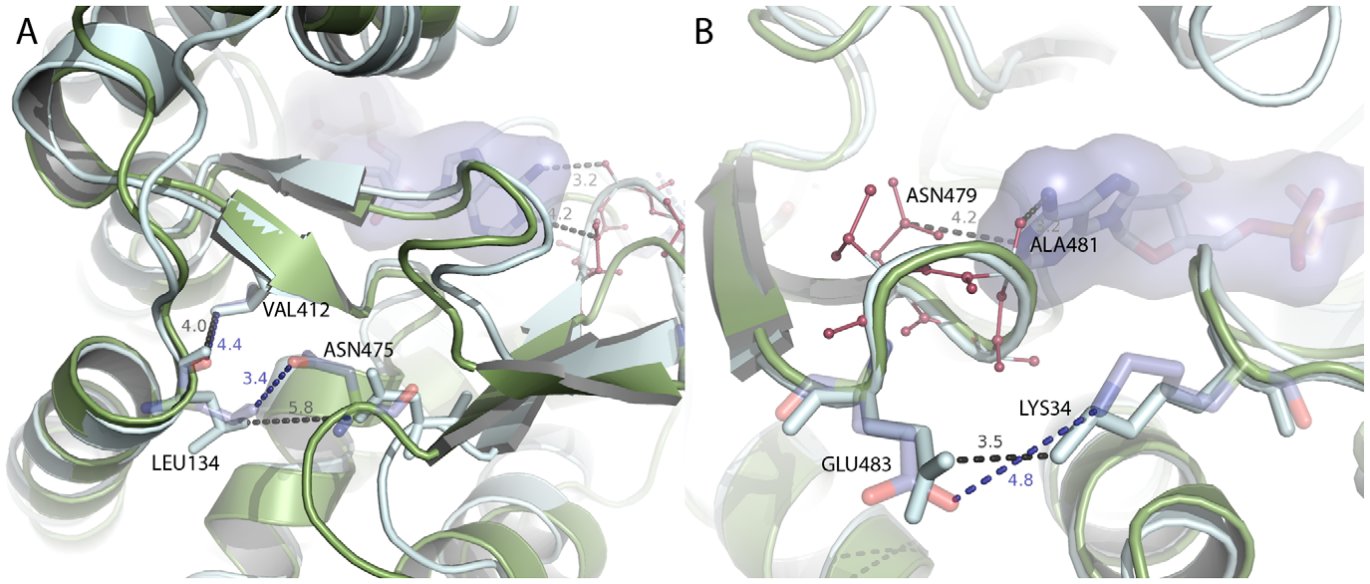
Local conformational changes in the equatorial domain upon nucleotide binding. X-ray structures of **t** and **r″** are shown in green and cyan colors, respectively. (A) shows the opposite side of the binding site where nucleotide binding increases the distance between Leu134 and Asn475. The closer contact between Lys34 and Glu483 brought about by nucleotide binding is shown in (B). ADP is shown in stick and surface representation.

While the ensemble of static X-ray structures provides the differential atomic interactions between the two end points of the reaction cycle, the MD simulations can reinforce the findings based on X-ray and further complement this by providing the differential atomic interactions at an earlier phase in the transition. Consistent with the findings from the X-ray-based DCM, the areas of high density differential contacts are situated in the equatorial and intermediate domains (Figure 7). Twenty differential contacts are consistently identified (Table 2). Perhaps the most interesting of these are the interactions Val412-Asn475 and Leu134-Asn475 located near the lower hinge (Figure 8A). These interactions show similar average distance difference in the X-ray and MD distance matrices. Moreover, they are located in the equatorial domain, close to the lower hinge, making them potentially important for the interaction between the equatorial and intermediate domain.

**Table 2:** Differential atomic interactions upon nucleotide binding obtained from X-ray structures and MD simulations.

Interaction	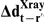	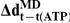	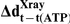
D83 K327	−30.4	−4.4	-
V177 R322	−21.9	−2.2	-
V412 N475	−1.8	−2.2	−1.8
L134 N475	−1.8	−2.0	−1.8
D179 K390	−2.7	−0.9	-
T176 V376	−1.9	−0.3	-
K7 D11	−0.8	−0.9	−0.8
L23 K75	−0.6	−0.9	-
V177 L400	−1.0	−0.3	-
L17 T101	−0.7	−0.6	-
N479 M488	−0.7	−0.2	−0.7
A85 V499	−0.5	−0.2	-
V412 I489	-	−0.8	−0.4
R231 E310	0.6	0.3	-
K65 L524	0.8	0.6	-
L31 T91	0.8	0.8	0.8
A84 Y506	1.0	0.7	1.0
V77 A507	-	0.8	0.9
V77 A92	2.2	1.0	-
V54 A78	3.5	1.4	2.2

The average difference in distance (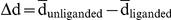) is shown for each of the three calculations; between X-ray **t** and **r″** (column 1), MD simulations of **t** with and without ATP (column 2), and X-ray **t** and ATP bound **t** conformers (column 3). Negative values (above mid rule) depict interactions present (or closer) in the nucleotide free state (unliganded), while positive values (below mid rule) depict interactions closer in the nucleotide bound state.

In the intermediate domain Asp179 and Lys390 connect helix M to three sheets (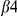, 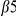, 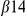) close to the apical domain in the **t** forms. These residues are 0.9 Å closer in the apo simulations than in the holo ones. This is consistent with the X-ray DCMs, which shows that Lys390 interacts with Thr48 in the equatorial domain.

The distance matrices can also be helpful to probe the underlying mechanisms for the changes in fluctuation pattern of the equatorial domain (Figure 4A–B). Table 3 summarizes the average difference in atomic distances for residues identified to have altered fluctuations. Of particular interest is Arg421 and Asn475 which show higher RMSF values in the holo simulations. Arg421 shows a tighter binding to Gly471 in the apo simulations than in the holo ones (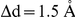). This is consistent with X-ray data, although the difference is about 1 Å smaller (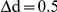 and 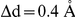). Perhaps the most evident difference affects Asn475 which in the MD simulations appears 2.2 Å closer to Val412, 2.0 Å closer to Leu134, and 1 Å closer to Ala413. These findings are again consistent with the differences found in the X-ray structures; i.e. 1.8, 1.8, and 0.8 Å for the **t**-**r″** difference, and 1.4, 2.5, and 0.7 Å for the **t**-**r** difference.

**Table 3:** Differential atomic interactions potentially involved in the stabilization of the equatorial domain.

Interaction	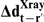	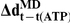	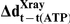
V412 N475	−1.8	−2.2	−1.4
L134 N475	−1.8	−2	−2.5
R421 G471	−0.5	−1.5	−0.4
V54 T89	0.5	−1.2	0.8
A413 N475	−0.8	−1	−0.7
L17 T101	−0.7	−0.6	−0.2
G53 T89	1.4	−0.5	0.8
V469 Y478	−0.1	−0.4	−0.2
Y485 M491	0.3	−0.3	−0.5
V54 S79	2.6	0.4	0.3
L17 I100	−0.2	0.5	−0.4
K28 V94	−0.5	0.6	0.1
V27 V56	0.2	0.7	0
V54 A78	3.5	1.4	1.7
K34 A480	0.4	2.2	0.7
K34 A480	0.4	2.2	0.7
K34 E483	0.8	2.7	1

See Table 2 for legend.

Interactions between 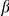
2-loop-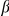
3 and 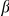
16-loop-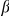
17, close to the ATP-binding site, are strengthened upon nucleotide binding which might explain the smaller RMSF values in this area (Figure 8B). The interaction Lys34-Glu483 is particularly affected; the average distance difference is 2.71 Å for the MD structures, while 0.8 and 1 Å for the X-ray structures. A significant difference is also found for Val54-A78 which are 1.4 Å closer in the holo simulation than in the apo simulations (3.5 and 1.7 Å for the X-ray differences).

**Figure 9:**
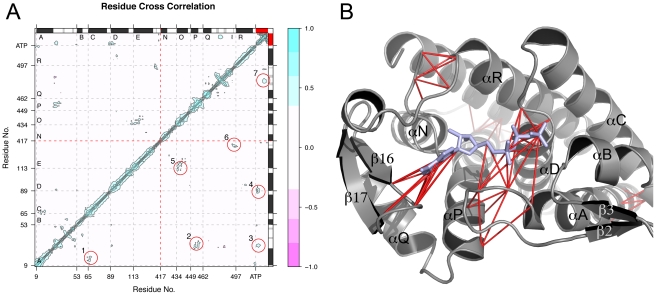
Residue correlated motions of the equatorial domain. (A) Cross-correlation map of the closed ATP bound simulation reveals residues that fluctuate in the same direction at the same time. The color scale runs from pink (anticorrelated motions) to blue (correlated motions). Red circles mark areas that are consistently found as correlated over all sets of 10 ns intervals of the trajectory. Secondary structure elements are indicated schematically with helices in black and strands in gray. (B) Mapping of the correlated residues onto the GroEL equatorial domain. Red lines link two correlated residues. ATP is colored blue.

Determining the residue cross correlation to investigate whether the motions of one residue are related to the motions of another can aid in deciphering the underlying mechanisms for the observed conformational transitions. We have previously reported the cross-correlation map for the entire GroEL subunit revealing global correlations [23]. In the present work we focus on the equatorial domain which has the advantage of revealing more detailed correlations around the binding site. The corresponding residue-residue correlation map for the equatorial domain is shown in Figure 9A, and highlights 7 off-diagonal correlated areas. These areas are consistently described as correlated throughout all subsets of the simulation (10, 20, and 50 ns intervals), and are the following: (1) residues Lys16-Gly20 (helix A) and Phe66-Asn68 (helix C), including two positively charged residues in helix A, and one negatively charged residue in helix C; (2) residues Gln454+Ile455+Asn458 (helix P) and Thr31-Gly33 (loop region between helix A and 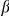
2); (3) The latter region is also correlated to the phosphates of ATP; (4) the phosphates of ATP and residues Gly89-Thr92 (helix D); (5) residues Asp115-Ile119+Ala123 and Asp435-Asn437+Ile440+Ala443; (6) Val411-Val412 (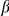
15) and Leu494-Thr497 (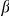
18) close to the lower hinge; and (7) the adenine ring of ATP and residues Asn479-Thr482 (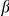
16-loop-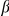
17 region).

## Discussion

### Extensive unbiased MD simulations

Several computational structural biology attempts have focused on the investigation of the ligand-induced conformational changes in GroEL [28], [30], [31], [33], [34]. Comparison of crystal structures at different stages of the functional cycle, NMA analysis and targeted MD simulations provide important information on the conformational space and motions associated to ligand-binding. Nevertheless, all-atom unbiased MD simulations have a superior potential to reveal the mechanisms involved in conformational transitions in proteins since using targeted MD simulations applying fictitious driving forces bias the motions toward the target and may drive the transitions along unrealistic deformations. However, GroEL has been considered to be beyond the analysis by unbiased MD simulations which have been held back due to the large size of the protein. Nevertheless, Sliozberg and Abrams have previously performed a 20 ns long unbiased MD simulation of one subunit, investigating the ATP-driven conformational changes [33]. This simulation manages to sample the transition from the **t** unbound, tense conformation to **r**, defined as a semi-relaxed conformation intermediate towards the open **r″** conformation. This transition includes a 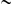 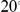 counterclockwise rotation of the apical domain along with closing of the nucleotide binding pocket, and was associated to the initial transition in a 2-stage activation by ATP [13], [30].

The **T** to **R** transition has been associated to the intra-ring cooperative allosteric response to ATP binding, since functionally the **R** conformer shows high affinity for ATP [13]. Following this reasoning Sliozberg and Abrams interpreted their results on the way the **r** structure would be concertedly transmitted to all seven subunits in the cis ring. In our present simulations of the isolated subunit, we sample close to the **r** structure (minimum deviation of 2.6 Å), and in addition, these long MD simulations (300 ns) provide so far unexplored insight into the ATP-induced transition from **r** all the way to a structure that is, according to the RMSd values, close to **r″** (minimum deviation of 5.8 Å). An almost full transition in the opposite direction, from **r″** to **t**, is revealed from a similar simulation of the ligand free apo state starting from the **r″** structure (minimum deviation of 5.1 Å). This transition would correspond to the conversion occurring in the trans ring, which functionally is accompanied by ejection of the hydrolysis product ADP and the co-chaperonin GroES. We thus show for the first time with unbiased MD simulations that the conformational transitions in the GroEL oligomer are favored by the intrinsic behavior of the isolated GroEL subunit. The predictive power of the present study is reinforced by the multiple 100 ns long MD simulations.

The conformer plots and the fluctations along the simulations also reveal that the **r** conformation is sampled both in the presence and absence of ATP. Thus, our results further add to the accumulating experimental and theoretical proofs of the pre-existing equilibrium between inactive and active states (see e.g. [52]–[56]). A similar trend was also detected in a recent fluorescence study where the authors were able to measure the fraction of molecules in the **T** and **R** state of GroEL with increasing ATP concentrations [57]. They determined that about 50% of the molecules were in the **T** state even with high ATP concentrations, indicating a constant cycling between the two conformations. Moreover, Chaudhry et al. showed that the inherent motions of the unliganded GroEL were “biased along the transition pathway that leads to the folding-active state” [58]. X-ray structures of the nonhydrolyzable ATP analog 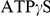, in which the domain rotations are not observed [9], give means to further support a **t** to **r** cycling.

These unbiased simulations also show that the open structure, similar to the **r″** structure, is attained for the isolated subunit, just in the presence of ADP, and without the need of the co-chaperonin GroES. It is accepted that binding of GroES to the heptameric cis ring with the subunits in the **r** conformation is the main functional determinant for the induction of the **r″** conformer, triggering the encapsulation of bound protein substrate into the cavity. Our results thus support that the subunit conformational changes along the functional cycle of GroEL, as revealed by X-ray crystallography and cryoelectron microscopy, are largely intrinsic to the 3D structure (sequence-structure-dynamics) of the subunit (intramolecular), though stabilization of intermediate conformations should be associated to intersubunit or interprotein (intermolecular) interactions in the GroEL-GroES oligomers.

Isolating a monomer from its natural biological assemblage may result in an altered dynamical behavior. However our results indicate that the dominant motion of a single GroEL subunit resembles that occurring in the ensemble of GroEL complex crystal structures. Thus, the dynamics of the subunit alone seems to be reflected in the larger biological assembly. This is seen by the RMSd analysis of the trajectories (Figure 3), as well as in the comparison between the X-ray and MD derived PCs (root mean squared inner product = 0.73) (Figure S13). The dominance of this intrinsic motion might be related to the relatively small inter-subunit contact area (6.6%, or 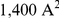) [8], which is comparatively low for an oligomeric protein [59], [60]. Nevertheless, each of the 7 apical domains interacts with two neighboring apical domains, as well as with one intermediate domain in the initial closed **T** form [8]. This arrangement imposes constraints on the motion of the subunit, though at present to an unknown degree. It is therefore expected that, for similar timescales, simulating the entire GroEL assembly would lead to a more restricted conformational sampling of the subunit which would most likely be more consistent with the timescale upon which these conformational transitions are thought to occur [15].

### Conformational changes

Sliozberg and Abrams described that MgATP binding to the subunit in the **t** conformation initiates a conformational change mostly due to the strong interaction of 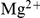 and Asp398 [33]. This interaction induces a series of H-bond ruptures (such as the Asp155-Arg395 salt bridge) and H-bond formations that were described in an induced-fit, cascade-like way [61]. We were however unable to reproduce this pattern of interactions. As seen in the conformer plots (Figure 2) the effect of ATP binding is rather described by causing a shift of conformational equilibria towards the **r″** conformation, with the peculiarity that in this case the ATP-bound conformation corresponds to the open conformation of the subunit, while usually ligand binding leads to a closed structure for the majority of proteins. In fact, in GroEL, ATP binding leads to a closed and less dynamic equatorial-domain structure but open and more dynamic (**r** and **r″**) subunit structure.

In the case of preexisting equilibria where the ligand binds selectively to an “active” conformation the energy barrier between the conformations in equilibrium should be low [52], and binding should not bring a high entropic penalty, as certainly seems to be the case for the GroEL subunit (Figure 6). Further experimental support for low energy barriers is also found for Hsp70 proteins [62]. Moreover, a thermodynamic study on nucleotide binding to GroEL provided a positive entropy change for the binding of ATP at temperatures higher than 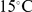 [63]. Notwithstanding the fact that these measurements were performed on the complete oligomer, it is noteworthy to relate the trend in entropy change to that found for the isolated monomer.

### Detailed changes

Computational studies employing advanced procedures in conjunction with NMA and brownian dynamics have probed allosteric networks in the GroEL system [31], [37], [39], [40]. They have been able to determine and highlight a large set of residues responsible for the transitions in the GroEL complex. Lys80-Asp359, Asp83-Lys327, Arg58-Glu209, Pro33-Asn153, and Gly257-Arg268 are among several intra-subunit interactions that have been identified to be important during the conformational transitions in GroEL [31], [39]. Of these, Arg58, Asp83, Gly209, and Lys327 were also highlighted by Tehver et al. [40], and shown to be highly conserved [64].

Our study of the MD trajectories and the large amount of X-ray data reinforces these studies as we identify many of the same interactions and residues. Moreover, we identify several other atomic differential interactions brought about by ATP-binding which have not previously been detected. Of particular interest is the tight binding between Lys34 and Glu483 in the presence of ligand (Figure 8A), which is captured in both the MD simulations and the X-ray crystallographic data. This effect appears as an important rearrangement induced by ATP-binding. These charged residues are situated close to the nucleotide binding site in two separate loops (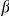
16-loop+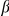
17 and 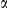
A-loop-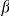
2), which movements interestingly are highly correlated to ATP; residues Asn479-Thr482 to the adenine ring, and Thr31-Gly33 to the phosphates of ATP. The correlation between these areas and ATP is likely to contribute to the stabilization of these loops as observed by fluctuation calculations (Figure 4), which in turn might aid in the salt-bridge formation between Lys34 and Glu483 (Figure 8B).

More peripheral to the ATP-binding site in the equatorial domain, close to the lower hinge, we observe weaker contacts between Leu134-Asn475, Arg421-Gly471, and Val412-Asn475 (Figure 8A). This is possibly due to rearrangements of helix N upon nucleotide binding which disrupts the initial interactions found in the **t** conformers and along our MD simulations of the unbound **t** forms. Moreover, the DCM analysis pinpoint an altered interaction pattern for helix C in the equatorial domain, possibly explaining the movements of helices A+C in the PCA analysis of X-ray structures. Among these interactions, hydrophobic amino acids are a common denominator; Ala84-Tyr506, Val77-Ala92, Val54-Ala78, Val77-Ala507 all show a tighter binding in the nucleotide bound conformers. Experimentally, this core of hydrophobic amino acids has been shown to be important, and e.g. the Ala92Thr protein vaiant shows a low ATPase activity [65]. Interestingly, the MD simulations also capture the rupture of the Asp179-Lys390 contact within the intermediate domain, and the formation of Arg231-Glu310 contact in the apical domain upon ATP-binding, both consistent with X-ray data.

Realizing that a deep understanding of the function of GroEL as a molecular machine certainly requires analysis of inter-subunit and inter-ring interactions in the oligomer, MD simulations of the complete GroEL complex would be required to probe the full effects of ATP-binding. While these are expected to reveal essential details on the allosteric regulation of GroEL function, 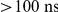 multisubunit simulations of a system of 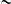
600.000 atoms are a very computationally-demanding task. Regardless of the capacity of GroEL to assemble into oligomeric structures, various studies have also shown the surprising facility to obtain stable folded monomers of GroEL by a variety of means [43]–[47], thay might display a weak chaperone activity of the GroEL monomer [46].

In the present work we have attained detailed and statistically proven results from MD simulations of the isolated GroEL subunit, which have highlighted the importance of considering the proper dynamics and response of the subunit in the context of the large scale transitions in the GroEL complex. Since the equatorial domain holds the ATP binding site, it constitutes the epicenter of the conformational changes in the GroEL complex. By paying extra attention to the detailed mechanisms in this region of the protein during the conformational transitions observed along the simulations we have been able to map important intramolecular rearrangements which potentially are a prerequisit for the intermolecular transitions to occur.

## Materials and Methods

### Structural analysis

All available GroEL structures were collected from the RCSB protein databank [66]. Six structures were omitted due to large amount of missing coordinates (PDB-codes: 2CGT, 1GR5, 1IOK, 2C7C, 1GRL, 3CAU). A total of 21 crystal structures (287 GroEL subunits) were kept for further analysis (PDB-codes: 1PCQ, 1PF9, 1SVT, 3C9V, 1AON, 1GRU, 1MNF, 1XCK, 2C7D, 2NWC, 3E76, 2EU1, 1SS8, 1SX3, 1J4Z, 1KPO, 2C7E, 1KP8, 1OEL, 1WE3, 1WF4). Structural superposition was performed on the invariant “core” as defined by Grant et al. [49]. These structures were collected and analyzed using the Bio3D package [49].

### Principal component analysis

The calculation of the PCA modes involves two main steps; (1) the calculation of the covariance matrix, 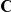, of the positional deviations, and (2) the diagonalization of this matrix [67], [68]. The 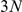 dimensional covariance matrix is calculated based on an ensemble of protein structures, and the elements of 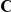 are defined as
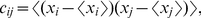
(1)where 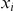 and 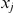 are atomic coordinates and the brackets denote the ensemble average. The diagonalization of the symmetric matrix 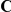 involves the eigenvalue problem
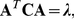
(2)where 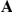 is the eigenvectors and 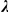 the associated eigenvalues.

Our PCA calculations were based on the C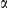 coordinates of the ensemble of the 287 GroEL crystal structures and was performed with the Bio3D package [49]. The X-ray and MD conformers were projected into the sub-space defined by PC1 and PC2, where the maximum variation of the conformational distribution was observed. Plotting these projections results in ‘conformer plots’ which displays a low dimensional representation of the conformational change in terms of the two principal components.

### Molecular dynamics simulations

All-atom MD simulations of the closed and open forms of the isolated GroEL subunit were performed both with and without bound nucleotide (designated holo and apo, respectively). The atomic models were prepared from the high-resolution crystal structures with PDB codes 1XCK chain A [69] and 1SVT chain A [58], for the closed and open forms, respectively. MgATP coordinates for the closed holo (with nucleotide) simulation was collected from the crystal structure with PDB id 1KP8 chain A [70]. All atomic models were prepared with Amber10 [71] and the corresponding Amber03 forcefield [72], [73]. ATP and ADP parameters were obtained from Meagher et al. [74]. For each of the simulations, the protein was solvated in a periodic truncated octhahedron box with TIP3 water molecules [75], providing 16 Å of water between the protein surface and the periodic box edge. The solute was minimized for 10,000 steps, followed by 10,000 steps of minimization of the entire system. The protein was then heated to 100 K with weak restraints for 100 ps, and to 300 K in 200 ps. 2 ns of equilibration with constant pressure and temperature (NPT) of the system was performed prior to the production run in order to ensure correct density. The production runs were performed with constant volume and energy (NVE) with a 1 fs time step, using SHAKE constraints on hydrogen-heavy atom bonds.

A total of 14 MD simulations of the GroEL subunit were carried out. Of these, we performed four 300 ns long simulations: (A) closed **t** unbound, (B) open **r″** unbound, (C) closed **t** ATP-bound, and (D) open **r″** ADP-bound. Additionally ten 100 ns long simulations of the closed **t** form were performed: 5 (E-J) unliganded, and 5 (K-P) ATP-bound. Simulations (D) and (K) consist of the 0–100 ns interval of simulations (A) and (C), respectively.

### Difference contact maps

Distance matrices were calculated between all 3856 pairs of atoms in order to monitor the atomic interactions (between residues at least four residues apart in sequence) [49]. The distance matrix was issued to residue grouping by only considering the minimal atomic distance between the residue pairs. Residue pairs closer than 4 Å are assumed to be in contact, and constitute the contact matrix for one particular conformation. Contact matrices were calculated for 28 closed (T) apo subunits (PDB code: 1XCK, 1SS8, 1OEL), 28 closed (T) ATP bound subunits (PDB code: 1KP8, 1SX3) and 28 open (R″) ADP bound subunits (PDB codes: 1AON, 1SVT, 1SX4, and 1PF9), and 6000 snapshots obtained from the last 50 ns of the 12 independent MD simulations on the closed GroEL subunit (6 apo and 6 holo). Only contacts with at least 50% occupancy and an average distance difference of 0.5 Å were considered. The difference of two contact maps (DCM), i.e. difference between apo and holo, then defines side-chain contacts which exist in one form, but not in the other.

### Additional analysis of simulated data

Clustering analysis, correlation maps, entropy calculations, and hydrogen bond analysis were performed with the the ptraj module of AmberTools [71]. Clustering was performed on the MD conformers using the average-linkage clustering algorithm [76]. Cross-correlation calculations were performed on several subsets of the closed ATP bound simulations (10, 20, and 50 ns intervals) in order to obtain areas of consistent correlations. All figures of GroEL are made in Pymol [77].

## Supporting Information

Figure S1 Statistics of projections of the MD conformers onto the first principal component. Each bar represents the projections (mean, median, or the last frame) of the simulations (only the 50–100 ns interval) onto the first principal component. The 6 apo and 6 holo simulations are depicted with grey and black bars, respectively. The full projections are provided in Figure 4.
(EPS)

Figure S2 Residual fluctuations of the closed unbound and ATP bound simulations for the intermediate and apical domains. Black bars show the RMSF values obtained from the ATP bound simulations, while red dots represent the unbound simulations. Binding site residues are depicted green, and the residues having the most significant changes are marked with labels. RMSF values are shown for the intermediate domain in (A) (134-190, 377-408), and apical domain (B) (191-376).
(EPS)

Figure S3Difference contact maps between t, r, and r″ conformers. Difference in side chain contacts are mapped for the X-ray structures (A-E), and MD structures (F). Two residues are assumed to be in contact if the minimal distance between them is 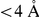. Only contacts existing in at least half of the structures are considered. Green triangles represent contacts present in the unliganded forms and not in the liganded forms. While blue dots represents contacts which are present in the liganded forms, but not in the unliganded forms. Orange lines display residues in which a change in contact partner has been detected. Red circles depict contacts which are found in both the X-ray and MD analysis. Secondary structure elements are indicated schematically with helices in black and strands in gray.(EPS)

Figure S4 Overlap map of the X-ray and MD PCs. Squared overlap between pairs of eigenvectors obtained from X-ray and MD PCA are shown. Dark grey corresponds to values close to 1 (high similarity) while white depict overlap close to 0 (orthogonal). Root mean squared inner product (RMSIP) is provided according to ref. [78].
(EPS)

Video S1 Time-dependent conformer plot of the 300 ns ATP-bound simulation. Projection of X-ray (contoured grey dots) and MD (blue) structures (along the simulation time) onto the two most significant principal planes defined by 287 GroEL experimentally obtained structures. The distribution of MD structures is depicted with density shaded blue points. Orange, red, and green dots represent the closed (**t**), semi-relaxed (**r**), and open (**r″**) X-ray structures, respectively. Gray dashed line depicts the average pathway of the conformational sampling.
(AVI)

## References

[1] Georgopoulos CP, Hohn B (1978). Identification of a host protein necessary for bacteriophage morphogenesis (the groe gene product).. Proc Natl Acad Sci USA.

[2] Houry WA, Frishman D, Eckerskorn C, Lottspeich F, Hartl FU (1999). Identification of in vivo substrates of the chaperonin groel.. Nature.

[3] Kerner MJ, Naylor DJ, Ishihama Y, Maier T, Chang HC (2005). Proteome-wide analysis of chaperonin-dependent protein folding in escherichia coli.. Cell.

[4] Fayet O, Ziegelhoffer T, Georgopoulos C (1989). The groes and groel heat shock gene products of escherichia coli are essential for bacterial growth at all temperatures.. J Bacteriol.

[5] Stan G, Brooks BR, Lorimer GH, Thirumalai D (2006). Residues in substrate proteins that interact with groel in the capture process are buried in the native state.. Proc Natl Acad Sci USA.

[6] Shtilerman M, Lorimer GH, Englander SW (1999). Chaperonin function: folding by forced unfolding.. Science.

[7] Lin Z, Madan D, Rye HS (2008). Groel stimulates protein folding through forced unfolding.. Nat Struct Mol Biol.

[8] Braig K, Otwinowski Z, Hegde R, Boisvert DC, Joachimiak A (1994). The crystal structure of the bacterial chaperonin groel at 2.8 å.. Nature.

[9] Boisvert DC, Wang J, Otwinowski Z, Horwich AL, Sigler PB (1996). The 2.4 å crystal structure of the bacterial chaperonin groel complexed with atp gamma s.. Nat Struct Biol.

[10] Xu Z, Horwich AL, Sigler PB (1997). The crystal structure of the asymmetric groel-groes-(adp)7 chaperonin complex.. Nature.

[11] Horovitz A, Willison KR (2005). Allosteric regulation of chaperonins.. Curr Opin Struct Biol.

[12] Horwich AL, Farr GW, Fenton WA (2006). Groel-groes-mediated protein folding.. Chem Rev.

[13] Ranson NA, Farr GW, Roseman AM, Gowen B, Fenton WA (2001). Atp-bound states of groel captured by cryo-electron microscopy.. Cell.

[14] Ranson NA, Clare DK, Farr GW, Houldershaw D, Horwich AL (2006). Allosteric signaling of atp hydrolysis in groel-groes complexes.. Nat Struct Mol Biol.

[15] Rye HS, Roseman AM, Chen S, Furtak K, Fenton WA (1999). Groel-groes cycling: Atp and nonnative polypeptide direct alternation of folding-active rings.. Cell.

[16] Rye HS, Burston SG, Fenton WA, Beechem JM, Xu Z (1997). Distinct actions of cis and trans atp within the double ring of the chaperonin groel.. Nature.

[17] Fridmann Y, Kafri G, Danziger O, Horovitz A (2002). Dissociation of the groel-groes asymmetric complex is accelerated by increased cooperativity in atp binding to the groel ring distal to groes.. Biochemistry.

[18] Karplus M, McCammon JA (2002). Molecular dynamics simulations of biomolecules.. Nat Struct Biol.

[19] Skjaerven L, Hollup S, Reuter N (2009). Normal mode analysis for proteins.. J Mol Struct THEOCHEM.

[20] Bahar I, Rader AJ (2005). Coarse-grained normal mode analysis in structural biology.. Curr Opin Struct Biol.

[21] Ma J (2005). Usefulness and limitations of normal mode analysis in modeling dynamics of biomolecular complexes.. Structure.

[22] Yang LW, Chng CP (2008). Coarse-grained models reveal functional dynamics–i. elastic network models–theories, comparisons and perspectives.. Bioinform Biol Insights.

[23] Skjaerven L, Martinez A, Reuter N (2011). Principal component and normal mode analysis of proteins; a quantitative comparison using the groel subunit.. Proteins.

[24] Marques O, Sanejouand Y (1995). Hinge-bending motion in citrate synthase arising from normal mode calculations.. Proteins.

[25] Cui Q, Li G, Ma J, Karplus M (2004). A normal mode analysis of structural plasticity in the biomolecular motor f(1)-atpase.. J Mol Biol.

[26] Mouawad L, Perahia D (1996). Motions in hemoglobin studied by normal mode analysis and energy minimization: evidence for the existence of tertiary t-like, quaternary r-like intermediate structures.. J Mol Biol.

[27] Ma J, Karplus M (1998). The allosteric mechanism of the chaperonin groel: a dynamic analysis.. Proc Natl Acad Sci USA.

[28] Zheng W, Brooks BR, Thirumalai D (2007). Allosteric transitions in the chaperonin groel are captured by a dominant normal mode that is most robust to sequence variations.. Biophys J.

[29] Keskin O, Bahar I, Flatow D, Covell DG, Jernigan RL (2002). Molecular mechanisms of chaperonin groel-groes function.. Biochemistry.

[30] Ma J, Sigler PB, Xu Z, Karplus M (2000). A dynamic model for the allosteric mechanism of groel.. J Mol Biol.

[31] Hyeon C, Lorimer GH, Thirumalai D (2006). Dynamics of allosteric transitions in groel.. Proc Natl Acad Sci USA.

[32] de Groot BL, Vriend G, Berendsen HJ (1999). Conformational changes in the chaperonin groel: new insights into the allosteric mechanism.. J Mol Biol.

[33] Sliozberg Y, Abrams CF (2007). Spontaneous conformational changes in the e. coli groel subunit from all-atom molecular dynamics simulations.. Biophys J.

[34] Abrams CF, Vanden-Eijnden E (2010). Large-scale conformational sampling of proteins using temperature-accelerated molecular dynamics.. Proc Natl Acad Sci USA.

[35] Brocchieri L, Karlin S (2000). Conservation among hsp60 sequences in relation to structure, function, and evolution.. Protein Sci.

[36] Stan G, Thirumalai D, Lorimer GH, Brooks BR (2003). Annealing function of groel: structural and bioinformatic analysis.. Biophys Chem.

[37] Chennubhotla C, Bahar I (2006). Markov propagation of allosteric effects in biomolecular systems: application to groel-groes.. Mol Syst Biol.

[38] Lu HM, Liang J (2009). Perturbation-based markovian transmission model for probing allosteric dynamics of large macromolecular assembling: a study of groel-groes.. PLoS Comput Biol.

[39] Yang Z, Majek P, Bahar I (2009). Allosteric transitions of supramolecular systems explored by network models: Application to chaperonin groel.. PLoS Comput Biol.

[40] Tehver R, Chen J, Thirumalai D (2009). Allostery wiring diagrams in the transitions that drive the groel reaction cycle.. J Mol Biol.

[41] Caves L, Evanseck J, Karplus M (1998). Locally accessible conformations of proteins: Multiple molecular dynamics simulations of crambin.. Protein Sci.

[42] Clarage JB, Romo T, Andrews BK, Pettitt BM, Phillips GN (1995). A sampling problem in molecular dynamics simulations of macromolecules.. Proc Natl Acad Sci USA.

[43] Lissin NM, Venyaminov SY, Girshovich AS (1990). (mg-atp)-dependent self-assembly of molecular chaperone groel.. Nature.

[44] Horovitz A, Bochkareva ES, Girshovich AS (1993). The n terminus of the molecular chaperonin groel is a crucial structural element for its assembly.. J Biol Chem.

[45] Makino Y, Taguchi H, Yoshida M (1993). Truncated groel monomer has the ability to promote folding of rhodanese without groes and atp.. Febs Lett.

[46] Taguchi H, Makino Y, Yoshida M (1994). Monomeric chaperonin-60 and its 50-kda fragment possess the ability to interact with non-native proteins, to suppress aggregation, and to promote protein folding.. J Biol Chem.

[47] Horowitz PM, Hua S, Gibbons DL (1995). Hydrophobic surfaces that are hidden in chaperonin cpn60 can be exposed by formation of assembly-competent monomers or by ionic perturbation of the oligomer.. J Biol Chem.

[48] White Z, Fisher K, Eisenstein E (1995). A monomeric variant of groel binds nucleotides but is inactive as a molecular chaperone.. J Biol Chem.

[49] Grant BJ, Rodrigues APC, ElSawy KM, McCammon JA, Caves LSD (2006). Bio3d: an r package for the comparative analysis of protein structures.. Bioinformatics.

[50] Harris SA, Gavathiotis E, Searle MS, Orozco M, Laughton CA (2001). Cooperativity in drug-dna recognition: a molecular dynamics study.. J Am Chem Soc.

[51] Clarage J, Romo T, Andrews B, Pettitt B, Phillips G (1995). A sampling problem in molecular-dynamics simulations of macromolecules.. Proc Natl Acad Sci USA.

[52] Teilum K, Olsen JG, Kragelund BB (2009). Functional aspects of protein flexibility.. Cell Mol Life Sci.

[53] Boehr DD, Nussinov R, Wright PE (2009). The role of dynamic conformational ensembles in biomolecular recognition.. Nat Chem Biol.

[54] del Sol A, Tsai CJ, Ma B, Nussinov R (2009). The origin of allosteric functional modulation: multiple pre-existing pathways.. Structure.

[55] Kern D, Zuiderweg ERP (2003). The role of dynamics in allosteric regulation.. Curr Opin Struct Biol.

[56] Cui Q, Karplus M (2008). Allostery and cooperativity revisited.. Protein Sci.

[57] Frank GA, Goomanovsky M, Davidi A, Ziv G, Horovitz A (2010). Out-of-equilibrium conformational cycling of groel under saturating atp concentrations.. Proc Natl Acad Sci USA.

[58] Chaudhry C, Horwich AL, Brunger AT, Adams PD (2004). Exploring the structural dynamics of the e.coli chaperonin groel using translation-libration-screw crystallographic refinement of intermediate states.. J Mol Biol.

[59] Miller S (1989). The structure of interfaces between subunits of dimeric and tetrameric proteins.. Protein Eng.

[60] Janin J, Miller S, Chothia C (1988). Surface, subunit interfaces and interior of oligomeric proteins.. J Mol Biol.

[61] Koshland DE, Némethy G, Filmer D (1966). Comparison of experimental binding data and theoretical models in proteins containing subunits.. Biochemistry.

[62] Taneva SG, Moro F, Velázquez-Campoy A, Muga A (2010). Energetics of nucleotide-induced dnak conformational states.. Biochemistry.

[63] Terada TP, Kuwajima K (1999). Thermodynamics of nucleotide binding to the chaperonin groel studied by isothermal titration calorimetry: evidence for noncooperative nucleotide binding.. Biochim Biophys Acta.

[64] Kass I, Horovitz A (2002). Mapping pathways of allosteric communication in groel by analysis of correlated mutations.. Proteins.

[65] Kovacs E, Sun Z, Liu H, Scott DJ, Karsisiotis AI (2010). Characterisation of a groel single-ring mutant that supports growth of escherichia coli and has groes-dependent atpase activity.. J Mol Biol.

[66] Berman H, Battistuz T, Bhat T, Bluhm W, Bourne P (2002). The protein data bank.. Acta Crystallogr D.

[67] Ichiye T, Karplus M (1991). Collective motions in proteins: a covariance analysis of atomic fluctuations in molecular dynamics and normal mode simulations.. Proteins.

[68] Amadei A, Linssen AB, Berendsen HJ (1993). Essential dynamics of proteins.. Proteins.

[69] Bartolucci C, Lamba D, Grazulis S, Manakova E, Heumann H (2005). Crystal structure of wild-type chaperonin groel.. J Mol Biol.

[70] Wang J, Boisvert DC (2003). Structural basis for groel-assisted protein folding from the crystal structure of (groel-kmgatp)14 at 2.0a resolution.. J Mol Biol.

[71] Case D, Darden T, Cheatham T, Simmerling C, Wang J (2008). Amber 10..

[72] Duan Y, Wu C, Chowdhury S, Lee M, Xiong G (2003). A point-charge force field for molecular mechanics simulations of proteins based on condensed-phase quantum mechanical calculations.. J Comput Chem.

[73] Lee M, Duan Y (2004). Distinguish protein decoys by using a scoring function based on a new amber force field, short molecular dynamics simulations, and the generalized born solvent model.. Proteins.

[74] Meagher K, Redman L, Carlson H (2003). Development of polyphosphate parameters for use with the amber force field.. J Comput Chem.

[75] Jorgensen W, Chandrasekhar J, Madura J, Impey R, Klein M (1983). Comparison of simple potential functions for simulating liquid water.. J Chem Phys.

[76] Shao J, Tanner S, Thompson N, Cheatham TE (2007). Clustering molecular dynamics trajectories: 1. characterizing the performance of different clustering algorithms.. J Chem Theory Comput.

[77] DeLano W (2010). The pymol molecular graphics system.. Schrödinger,.

[78] Amadei A, Ceruso MA, Nola AD (1999). On the convergence of the conformational coordinates basis set obtained by the essential dynamics analysis of proteins' molecular dynamics simulations.. Proteins.

